# Event and Comment

**Published:** 1911-08-15

**Authors:** 


### DENTAL REGISTER.

**Vol. LXV. August 15, 1911. No. 8**

### EVENT AND COMMENT.

NATIONAL DENTAL ASSOCIATION ANNUAL CONVENTION. The fifteenth annual meeting was held at Cleveland July 25th to 28th, inclusive, and as predicted it was probably the largest meeting of this Association ever held. There were over three thousand members and guests registered, but all were not dentists. It is probable that twenty-five hundred dentists attended the various meetings. As usual the clinics drew the largest numbers. The large room were they were held was crowded both days. The clinics were very good and a larger number materialized than is customary at such large meetings. They were mostly table clinics, although there were a considerable number of excellent chair clinics. The surgical clinics were not all made because of failure of the patients to keep their promises. Our space will not permit of even mentioning them, but there was a good representation of all the technical advances of the past year. There seemed to be a larger number of Bridge clinics than of any other thing. Some beautiful specimens were shown of removable pieces for the grinding teeth. There was not so many casting clinics as last year, although this department was well represented. Anesthesia by oxygenated nitrous oxide was prominent, and so were the pyorrhea enthusiasts. A great deal of interest centered about the children who had been used as a test to determine the result of dental interference and treatment on the mental activity of children taken from the Marion School of Cleveland. Twenty-seven children were cared for dentally only, and increased in efficiency on the average nearly one hundred percent. during the year. The methods of caring for the children and of making the tests for calculating the relative progress were shown and explained. The result is simply astonishing in its physical as well as its mental findings. It was stated that if similar work was carried on in every city in the United States, millions of dollars annually could be saved the tax payers.

One evening was devoted to hearing reports from the Oral Hygiene Committee, and another to perfecting a national organization of dentists and laymen for carrying on the oral hygiene propaganda. A temporary organization was perfected with Miss Cordelia L. O'Neil as temporary chairman and Dr. W. G. Ebersole as temporary secretary. The idea is to raise money independently of the National Association and push the work of oral hygiene as actively as possible.

THE PROGRAMME. Seventeen papers were read and discussed. As usual, some papers got plenty of time for adequate consideratoin while others had no discussion at all, even by those formally appointed to discuss them. Two papers were read by title only for lack of time. One paper took up two and one-half hours to read and had a half hours discussion. There was much displeasure expressed because of the conduct of the section meetings, and a resolution was passed that next year no paper shall occupy more than thirty minutes in reading and the formal discussers shall have ten minutes each and general discussers five minutes each. Such a rule will, of course, be objectionable in some cases, but it will be less injurious than permission of an essayist to take up more time than he is justly entitled to. It would be difficult to single out the most important papers, as every one will value a paper according as it expresses his ideals or contains matters of special interest to him. It was a great pleasure to listen to the well phrased address of Dr. S. G. Perry, of New York, on "Evolution in Dentistry." It was a fine historic review of dental progress by one who has seen the most active period of the development of our profession. The paper of Dr. Case on "Extraction in Orthodontia" was an elaborate presentation of his views, and was controversial in its whole attitude, taking issue with the well-known attitude of the Angle School of Orthodontists on this question. It was a masterly essay, but entirely too long for presentation at such a place. Dr. V. E. Barns made a valuable contribution on the subject of "Impactions and Preventive Treatment," but his paper got no adequate discussion.

Dr. R. Ottolengui read a paper on the "Ethics of Patents" which brought out a lively discussion. His paper was pointed at a justification of Dr. Taggart's so-called process patent, and naturally it brought out the opposition in rigorous fashion. We do not see what good can come from such aggitations, nor why this subject cannot be allowed to rest until the court has passed upon the validity of these patents, as it will probably do within a few months. Such affairs cannot be settled by argument and much bitterness and unbecoming conduct always results from a public discussion of them. The other papers read were most valuable contributions to the technical departments and space will not permit our commenting on them. The report of Dr. Ferris, Chairman of the "Research Committee," gave a detailed statement of the results of his labors during the past year in determining the chemical contents of the saliva. This is the kind of work the National Society ought to encourage; but we regret to say that no adequate encouragement has ever been extended to any one, either in the way of money to carry out the details of research or in bringing the results before the general profession. This valuable report was crowded out until near the close of the session, when there was neither time nor interested persons present to discuss it. May we not hope that the new constitution will provide suitable means for this most important branch of society work?

REVISION OF CONSTITUTION. A meeting of delegates from twenty-two State societies met the day before the national meeting and decided to recommend the re-organization of the National Association along the lines of the American Medical Association. The committee will draft a constitution and submit it to the various State societies for consideration and recommendations, and the matter of re-organization will be taken up at the next meeting to be held in Washington City in 1912. This will undoubtedly be a large piece of work as the several State societies are so variously situated that it will be difficult to secure a harmonious agreement. We trust the committee will be wise enough to draft a constitution that will meet with sympathetic consideration by a large number of states, as the success of the revision will depend wholly upon its acceptance by many State societies. It will be necessary for every State society to make some concessions, but the object in view is certainly of such general benefit that no state society should hesitate to lend its co-operation, even though it may introduce local problems difficult of solution. There certainly can be little doubt that a strong National Society can only be made by the co-operation of the state and local societies, and it can also be confidently predicted that the centralization of many state activities in a great organization will react in the most beneficial manner and prove helpful in strengthening the state and local organizations. We certainly have arrived at a period when something radical must be done to even conserve our present professional standards, and a wise disposition of this matter will undoubtedly assure future advancement in all our professional activities.

EVENTS OF THE NATIONAL MEETING. The next place of meeting will be in Washington City, the second week of September. The choice rested between Louisville and Washington as representatives of the Southern section. The time was deferred so that the temperature might be more favorable than the usual date. This should be a good meeting.

The officers elected were: Dr. A. R. Melendy, of Knoxville, Tenn., as President; Dr. L. P. Dotterer, of Charleston, S. C., Vice-President for the South; Dr. D. O. M. Le Cron, of St. Louis, Vice-President for the West; Dr. L. L. Barber, of Toledo, Vice-President for the East; Dr. Charles W. Rodgers, of Boston, Corresponding Secretary; Dr. H. C. Brown, of Columbus, Recording Secretary; Dr. H. B. McFadden, of Philadelphia, Treasurer. Dr. J. D. Patterson, Dr. C. J. Greaves and Dr. Victor C. Jones were elected to the executive council. Dis. H. J. Burkhart, Charles McManus, A. H. Peck, E. R. Warner and B. Holly Smith were elected to the executive committee.

CLEVELAND'S ENTERTAINMENT. It would be difficult indeed for us to express in words the appreciation heard on all sides of the tremendous work the Cleveland dentists must have done to take care of the convention, so thoroughly prepared was everything for their guests. The weather was ideal, the accommodations ample and good, the meeting places admirable and well conducted, the social entertainments for the ladies abundant and cordial, the general social entertainment of a boat ride on the lake for all guests most generous and delightful, and the innumerable smaller attentions of a personal nature made the entire week one of great pleasure and profit, laying us under great obligations for all these generous and thoughtful provisions for our comfort.

We learned that the local committee raised and expended nearly eight thousand dollars, and gave a lot of valuable time that we might enjoy all these comforts and benefits. Our cordial thanks are most sincerely extended and we earnestly hope that, while we came and partook of the feast with pleasure, our hosts may ever realize the deep pleasures of their bountiful and appreciated hospitality.

